# Input-Output Modeling for Urban Energy Consumption in Beijing: Dynamics and Comparison

**DOI:** 10.1371/journal.pone.0089850

**Published:** 2014-03-03

**Authors:** Lixiao Zhang, Qiuhong Hu, Fan Zhang

**Affiliations:** 1 State Key Joint Laboratory of Environmental Simulation and Pollution Control, School of Environment, Beijing Normal University, Beijing, China; 2 The World Bank, Washington DC, United States of America; DOE Pacific Northwest National Laboratory, United States of America

## Abstract

Input-output analysis has been proven to be a powerful instrument for estimating embodied (direct plus indirect) energy usage through economic sectors. Using 9 economic input-output tables of years 1987, 1990, 1992, 1995, 1997, 2000, 2002, 2005, and 2007, this paper analyzes energy flows for the entire city of Beijing and its 30 economic sectors, respectively. Results show that the embodied energy consumption of Beijing increased from 38.85 million tonnes of coal equivalent (Mtce) to 206.2 Mtce over the past twenty years of rapid urbanization; the share of indirect energy consumption in total energy consumption increased from 48% to 76%, suggesting the transition of Beijing from a production-based and manufacturing-dominated economy to a consumption-based and service-dominated economy. Real estate development has shown to be a major driving factor of the growth in indirect energy consumption. The boom and bust of construction activities have been strongly correlated with the increase and decrease of system-side indirect energy consumption. Traditional heavy industries remain the most energy-intensive sectors in the economy. However, the transportation and service sectors have contributed most to the rapid increase in overall energy consumption. The analyses in this paper demonstrate that a system-wide approach such as that based on input-output model can be a useful tool for robust energy policy making.

## Introduction

Cities account for the largest share of energy consumption and also provide concentrated opportunities for large energy savings [1]. This is particularly true in China, where cities accounted for 84% of China's total commercial energy consumption in 2006 [2]. By the end of 2012, urban population in mainland China reached 712 million, or 53% of the total population, rising from 26% in 1990 [3]. At the same time, the government is projecting the urbanization of an additional 350 million people–greater than the population of the entire United States–over the next 15 years [1]. Such large-scale urbanization would have huge implications for future energy demand and related environmental impacts.

The traditional way of analyzing urban energy consumption highlights only the direct energy consumption of end-user sectors, which normally consist of agricultural, industrial, transport, commercial, and residential sectors [2], [4]–[6]. However, urban economic systems are highly complex and integrated. Each economic sector not only consumes energy in a direct way in forms such as electricity, oil, coal, and natural gas, but also in an indirect way by consuming energy-intensive intermediate inputs produced by other sectors. Ignoring the linkage between sectors and focusing only on direct and final energy consumption would underestimate the amount of city-level energy consumption and undermine the efforts of energy savings.

A more holistic way of analyzing city energy consumption is to account for both direct and indirect energy consumption, the latter of which refers to energy usage that is embodied in the intermediate products and material that are then passed on to other sectors until they reach the final consumer. By focusing on embodied (direct and indirect) energy consumption, one can identify the drivers of the overall energy consumption and quantifies the amount of energy usage for which each sector is ultimately responsible [7]–[9]. The ratio of direct and indirect energy use can also provide essential information concerning a city's economic structure [10]–[12].

Input-output (I-O) technology-based embodiment analysis has recently become a popular method for benchmarking systems energy accounting, which could facilitate a deeper appreciation of each sector's total energy requirements, including both the direct, visible requirements and the indirect, hidden energy costs[13]–[27]. With the help of the Leontief inverse matrix (see more details in section 2.1), such an approach could account for the cumulative energy requirements of a sector regardless of the complexity and length of the production process [28]–[31]. Chen and his research group carried out an analysis of embodied resources and emissions at China's national level [32]–[34]. Liu et al [11] use 2007 input-output table to analyze embodied energy use in China's industrial sectors. At the city level, Zhou et al. carried out an embodied resource accounting analysis of Beijing's economy [35]; Liang et al. conducted a case study of Suzhou, involving the identification of key sectors [12].

As the capital city of China, Beijing exemplifies the rapid urbanization and economic growth that has occurred in China over the past two decades. Currently, Beijing is entering into a phase of extensive deindustrialization and economic restructuring, with the relocation of the Beijing Shougang Iron and Steel Plant from Beijing to Caofeidian, Tangshan a vivid example of such a strategy [5].

In this paper, we use an environmental input-output model to analyze the embodied energy consumption of Beijing from 1987 to 2007. The main aims of this paper are: (1) to find the variations of total energy consumption (directly and indirectly) for Beijing during the past 20 years alongside rapid urbanization, (2) to compare the sectoral distribution of embodied energy consumption between 1987 and 2007 with regard to economic structural changes, (3) to reveal the transmission mechanism of energy flow through the entire supply chain or among sectors. The rest of the paper is structured as follows: Section 2 provides an introduction of the environmental input-output method and data source. Section 3 presents the results and discussions. Concluding remarks are provided in Section 4.

## Methodology

### Environmental input-output model

The I-O method has been applied to embodied energy accounting many times over the past decades, without major changes in methodology [36]. In fact, some references from the 1970s cited in this paper read as if they had been written in twenty-first century [37]. Technically, an environmental input-output model has to be built through integrating the economy with its energy consumption by industrial sectors. Each sector in the economy uses primary energy as a direct energy input into their production process, which eventually gravitates towards final demand. Thus, every sector takes direct energy in the form of coal, natural gas and so on, and indirect energy through the embodied energy in inputs from other sectors. It should be noted that this method is based on some key assumptions and subject to certain constraints and limitations [9], [32], [38]. For instance, it is assumed that imported commodities (both domestic and foreign imports) have the same embodied energy intensity as local products. Certainly this is not accurate, but we need to work with such an assumption because we simply do not know the import structure for ports of origin outside of China [39]. Although the development of a multiregional input–output (MRIO) model can partially address this issue [40]–[42], it is nearly an impossible task to build 9 MRIO models covering domestic provinces and various countries in the world with regard to the data intensive feature of the MRIO model and the limited data availability.

With reference to [34] and [40], an environmental input-output table for the urban economy was built to integrate the economic activities and energy consumption or emissions (see table 1). The embodied energy analysis framework using the I-O table has been described extensively in the literature [8], [9], [17], [23], [32], [43]. In the following, only the most important aspects of this methodology are summarized. With regard to the inputs and outputs, the central balance equation is: 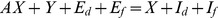
(1)where 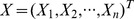 is the (n×1) vector of gross outputs for each economic sector i; 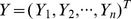 is the (n×1) vector of final demands with elements Y_i_; 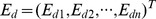 and 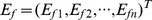 denotes the outputs exported to the domestic and foreign markets from sector i; Similarly, 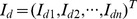 and 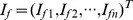 denote the imported inputs from the domestic and foreign markets to sector i. A is the technology coefficient matrix (n×n) with elements a_ij_, describing the amount of intermediate demand of output from the domestic sector i used by domestic sector j;

**Table 1:** Scheme of the environmental input-output table as an integration of urban economy and energy[35], [40].

Sector	Intermediate use				Final use			Import from domestic (I_d_)	Import from foreign (I_f_)	Total output (X)
	1	2	…	n	Final demand (Y)	Export to domestic (E_d_)	Export to foreign (E_f_)			
1	x_11_	x_12_		x_1n_	Y_1_	E_d1_	E_f1_	I_d1_	I_f1_	X_1_
2	x_21_	x_21_		x_21_	Y_2_	E_d2_	E_f2_	I_d2_	I_f2_	X_2_
…	…	…	…	…	…	…	…	…	…	…
n	x_n1_	x_n2_		x_n1_	Y_n_	E_dn_	E_fn_	I_dn_	I_fn_	X_n_
Value added	v_1_	v_1_	…	v_n_						
Total input (X)	X_1_	X_2_	…	X_n_						
Direct energy inputs	E_1_	E_2_		E_n_						

If E (1×n) denotes the direct energy inputs for each sector from the perspective of sectoral production, Ω (1×n) denotes the factor vector of the energy intensity for each sector, then 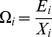
(2)


If ε (1×n) denotes the embodied energy demand per unit of production of each sector within the city, then 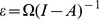
(3)where I represents the identity matrix, 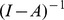 is the Leontief inverse.

We know E is the total direct energy consumption from the production perspective and E_i_ is the direct energy demand for production activities by different sectors. In contrast to the direct energy requirement E (E_i_), the indirect items of energy inputs are relative concepts. The term “indirect” can be defined in two ways depending on the context. For the whole city, it refers to any primary, secondary, or final energy consumed outside the boundary of the metropolitan area in order to produce energy, goods or services consumed by any entity, public or private, inside the metropolitan area [44]; For a certain sector i, however, indirect energy usage is associated with energy embodied in intermediate use from other sectors.

Therefore, for the whole city, we have reformulated the equation (1): 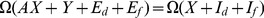
(4)


After several steps of transformation, we get, 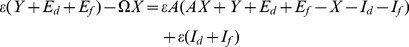
(5)


Since 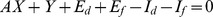 and 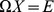, it can be proved that the indirect energy requirement for the entire urban system equals the embodied energy imported from outsides. 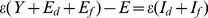
(6)


For a specific sector, the indirect energy necessary for output production can be described as: 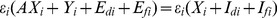
(7)


which can be further denoted as 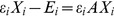
(8)


### Indicators of influence coefficient and response coefficient

As mentioned above, every sector takes direct energy from primary energy resources, and indirectly through the embodied energy in inputs from other sectors within the urban economic system. From an energy management perspective, it is important to understand the links between sectors in energy consumption. Therefore, the influence coefficient (IC) and response coefficient (RC) were used as indicators to identify the key sectors in energy transmission mechanism along the production chain. The IC refers to the effect induced on all sectors due to an increase of one unit of final demand in a particular sector. It reflects the backward linkage effect, i.e., the severity of impact in a sector that diffuses to all other sectors. The RC refers to the effect induced in one particular sector due to an increase in one unit of final demand from all sectors. It reflects the forward linkage effect, i.e., the amount a particular sector receives from all other sectors [31]. These two indicators have often been used to analyze industrial linkages. Based on traditional correlation theory, Rasmussen extended these indicators to the energy analysis of industrial sectors [45].

The IC and RC of energy consumption by industry sector can be derived as follows. If the demand increases by *k* percent, then the change in the embodied energy of the total final demand can be expressed as: 
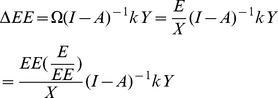
(9)


Where EE is the embodied energy consumption of total final demand, and ΔEE is the change in the embodied energy of the total final demand due to an increase in final demand. Let B = E/EE = (B_1_, B_2_ …B_i_), where B_i_ refers to the ratio of direct energy to embodied energy in the total final demand of sector i: 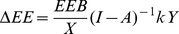
(10)

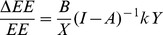
(11)


Let k = 1; then 
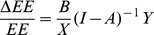
(12)


By diagonalizing the B and Y vectors and to let 
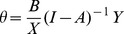
(13)where θ is the industrial linkage matrix of energy consumption by sector, of which the *ij*-th element is *B_i_m_ij_Y_j_*/*X_i_*, denoting the change of energy consumption in sector *i* when the demand of sector *j* increases by one percent, and *m_ij_* is the coefficient of the Leontief inverse matrix, i.e., the total demand required to produce one unit of product in sector *j*.

The RC is the sum of the row elements and can be expressed as: 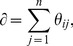
(14)which represents the percentage change in energy consumption by sector *i* when the demand of other sectors increases by one percent.

The IC is the sum of the column elements and can be expressed as: 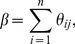
(15)which represents the percentage change in energy consumption by other sectors when the demand of sector *j* increases by one percent.

### Data source

The analysis in the paper is based on the input-output tables of the following years: 1987, 1990, 1992, 1995, 1997, 2000, 2002, 2005, and 2007, which are compiled by the Beijing Statistical Bureau [46]. The tables for 1987, 1990, 1992, and 1995 cover 33 economic sectors, and 40 sectors for 1997 and 2000, while the tables for 2005 and 2007 include 42 sectors. We aggregate the different sectors in different years into 30 sectors to keep these datasets consistent. The 30 sectors are then further divided into 5 more aggregated sector groups, i.e. agriculture (A), mining (B), manufacturing (C), construction (D) and services (E). The entries in the tables valued in the producers' prices are converted into 1987 constant prices using the GDP price index. The sectors' classification and sector codes are shown in Table 2. The direct energy consumption data for each sector are derived from the energy balance table (EBT) of the Beijing Statistics Yearbook for the corresponding years [47] (see Table S1 of appendix). For the use of energy data in the EIO framework, it is necessary to allocate all energy back to the primary carrier [48].

**Table 2:** Aggregated sectors and groups for input-output analysis [35], [40].

Group code	Group name	Sector code	Sector name
A	Agriculture	1	Agriculture
B	Mining	2	Mining and washing of coal
		3	Extraction of petroleum and natural gas
		4	Metal ore mining
		5	Nonmetal mineral mining
C	Manufacturing	6	Food industries
		7	Textiles
		8	Wearing apparel, leather, furs, feather and related production
		9	Sawmill products and furniture
		10	Paper products, printing, and recording media reproductions
		11	Electricity, steam, and hot water production
		12	Petroleum processing and coking
		13	Chemicals
		14	Nonmetallic mineral products
		15	Metal smelting and pressing
		16	Metal products
		17	General- and special-purpose machinery
		18	Transportation equipment
		19	Electric equipment and machinery
		20	Electronic and telecommunication equipment
		21	Instruments and other measuring devices
		22	Other manufactured products
D	Construction	23	Construction
E	Service	24	Transportation, storage, and postal services
		25	Business services
		26	Accommodation and food services
		27	Public services
		28	Professional, scientific, and technical services
		29	Finance and insurance
		30	Public administration and other sectors

## Results and Discussion

### Dynamic changes of energy consumption for whole urban economy of Beijing

**Figure 1:**
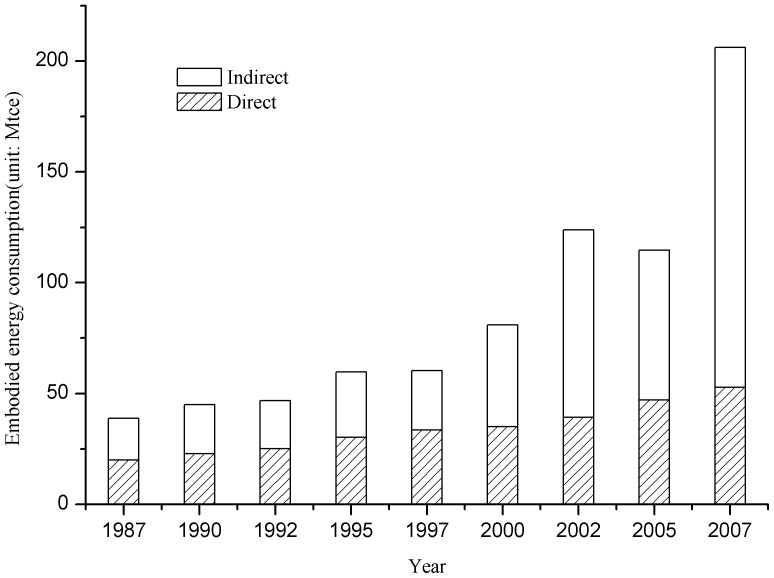
Embodied energy consumption of Beijing city from 1987 to 2007.

Figure 1 shows the direct and indirect energy demand of Beijing in the 9 years analyzed. With regard to the aggregate city-level energy consumption in Beijing, the total direct energy consumption increased by 2.64 times, rising from 20.31 Mtce in 1987 to 52.79 Mtce in 2007. This represents a 5% annual growth rate. Prior to 1997, direct energy consumption was always greater than indirect energy consumption. This trend, however, reversed after 2000, suggesting that Beijing has become more reliant on outside sources for energy demand. Regarding the increase of indirect energy consumption, there are two rapid growth periods: one from 1997 to 2002 and another from 2005 to 2007. The construction activities as a result of real estate development were, to a large extent, responsible for the rapid growth in indirect energy consumption, further illustrated by an embodied energy analysis of the construction sector in sections 3.2 and 3.3. To cope with the 1997 Asian financial crisis and to fuel domestic demand, real estate development had been encouraged by the government during 1997–2002. The preparation of 2008 Olympics Game contributed to the large wave of urban construction during 2005–2007. The boom and bust of real estate development over time in Beijing and in China in general is also worth mentioning. Specifically, the declining trend of indirect energy consumption during 2002–05 can be attributed to the real estate development suppression policies.

**Figure 2:**
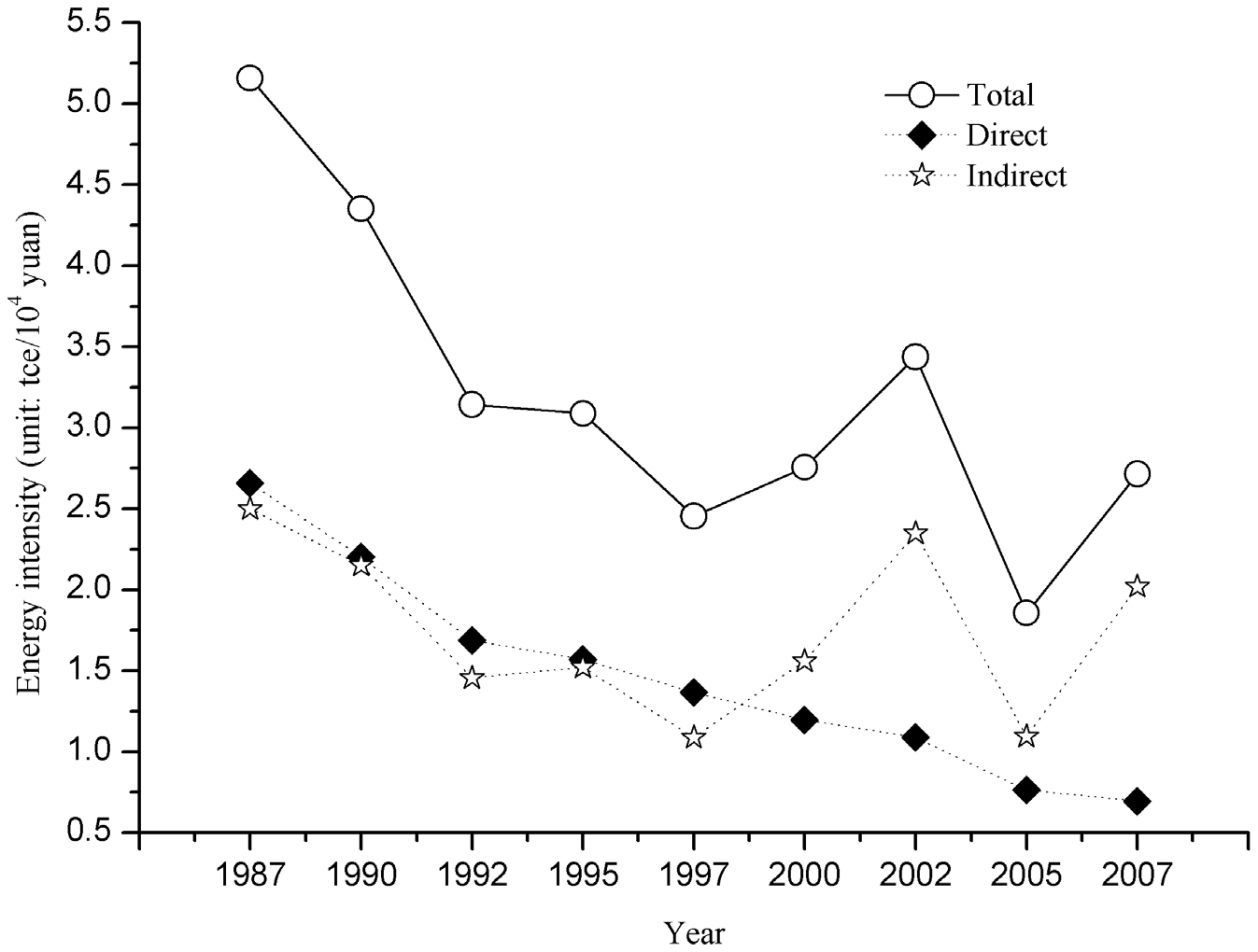
The changes of energy consumption intensity of Beijing city from 1987 to 2007.

Overall, the total embodied energy consumption of Beijing increased from 38.85 Mtce in 1987 to 206.2 Mtce in 2007 Mtce, representing a growth rate of 9% per year. Although total energy consumption has significantly increased over time, the energy intensity has, on the contrary, declined, with direct energy intensity decreasing from 2.66 tce/ten thousand yuan to 0.70 tce/ten thousand yuan, and embodied energy intensity decreasing by 52%, from 5.16 to 2.71 tce/ten thousand yuan, during the past 20 years (Figure 2).

In addition, it is interesting to note that the proportion of direct energy in embodied energy has declined from 52% to 26%, while that of indirect energy has increased from 48% to 76%. Since indirect energy demand for the whole city reflects how a city relies on outside sources, the increase of indirect energy use reveals the evolution of Beijing's economic structure, which will be discussed intensively in the later section of this paper. Imagine an urban economy in which direct energy makes up only about one quarter of consumption, where technology has been efficient for some time, and further reductions are likely to be less efficient.

### Comparison of sectoral energy consumption between 1987 and 2007

**Figure 3:**
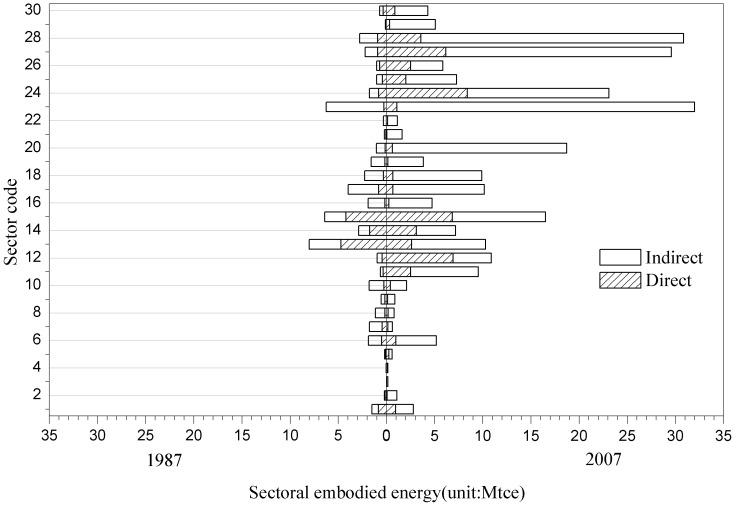
Sectoral distribution of embodied energy consumption between 1987 and 2007 in Beijing (see Table 2 for sector code definitions).

**Figure 4:**
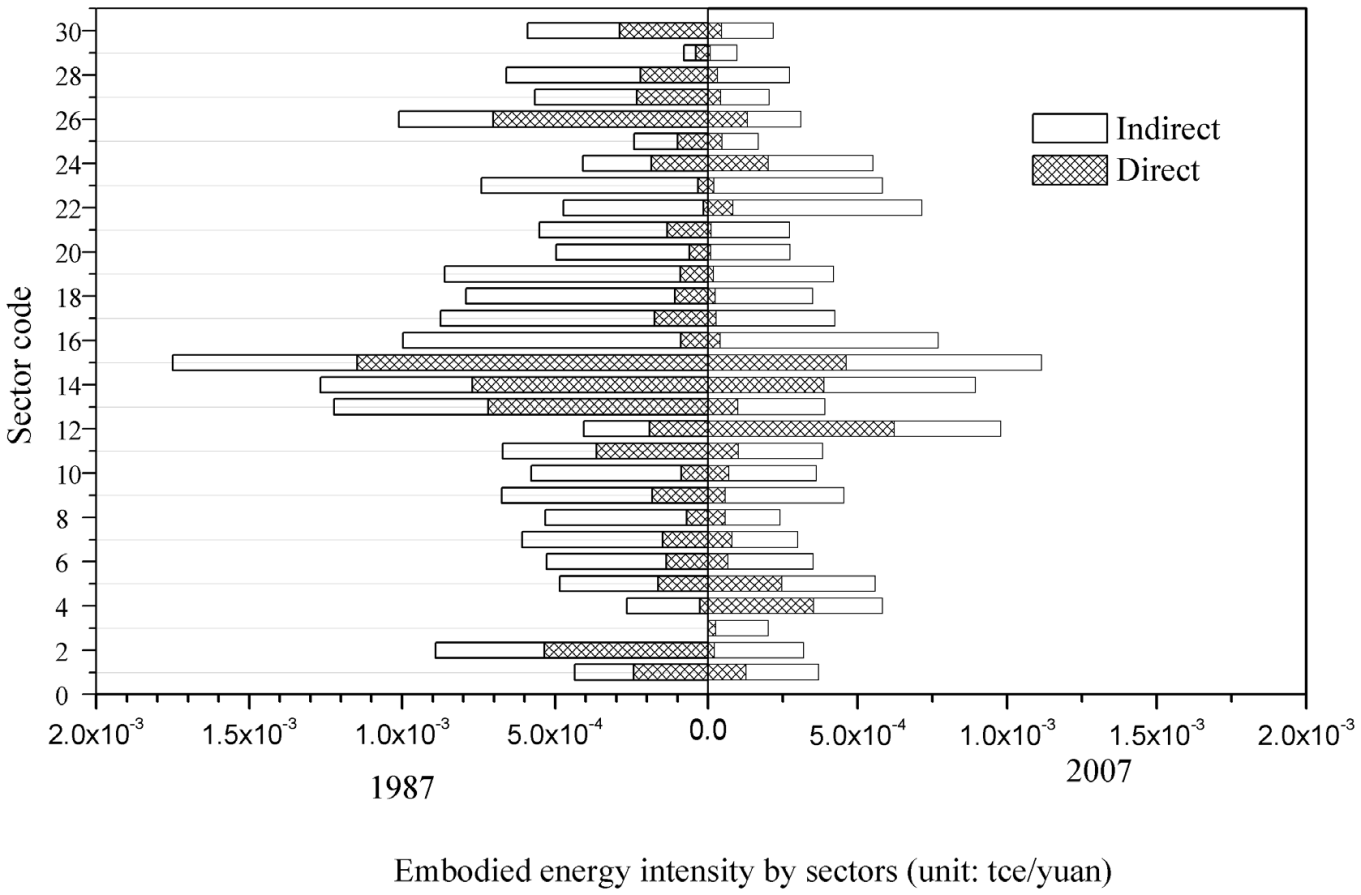
Embodied energy intensity by sector between 1987 and 2007 in Beijing (see Table 2 for sector code definitions).

Figure 3 compares sectoral embodied energy consumption in 1987 and that in 2007. Figure 4 illustrates the comparison results of sectoral energy intensity of these two years. Overall, the sectoral embodied energy consumptions were substantially higher while intensities were relatively lower in 2007 than their counterparts in 1987; Sectors in Group C, D, E account for most of the energy consumption in 1987 and 2007, while sectors in Group A and B consumed much less. Beijing lacks natural resources, demonstrated by the fact that all of the natural gas and crude oil consumed by the city, as well as 95% of the coal, 64% of the electricity, and 60% of the refined oil consumed are imported from outside [49], which explains why Group B is not a large energy user.

With regard to sector-by-sector comparison, substantial changes can be found in the distribution of direct and indirect energy consumption during this time period. For instance, both the largest direct and indirect energy consumers are in Group C in 1987, such as sector chemicals (No. 13), while in 2007 the largest direct energy consumer is the transport sector in Group E and the largest indirect energy consumer is in Group D, i.e., the construction sector; Some traditional manufacturing sectors in Group C decreased their direct energy consumption due to the downsizing of the sectors (e.g., textiles sector) or technology upgrade (e.g. chemical sector). There is no doubt that the current extensive deindustrialization and economic restructuring in Beijing have affected energy consumption in many ways. For example, the relocation of Beijing Shougang Iron and Steel Plant from Beijing to Caofeidian, Tangshan, Hebei province may have contributed to the changes of industrial business of sector No. 2 and No. 17 in Group C. Of all the sectors, the direct energy consumption of sector 24 (transportation, storage, and postal services) increased the most, by 7.61 Mtce, followed by sector 12 (petroleum processing and coking), with an increase of 6.48 Mtce, which are highly interrelated. The rapid growth of direct energy consumption in transportation and petroleum processing can be attributed to the rapid development of the transportation system in Beijing: the number of motor vehicles in Beijing increased 11.28 times, from 0.27 million vehicles to 3.1 million during this period.

As to the embodied energy consumption, the most dramatic changes occurred in the downstream sectors, such as the construction sector (No. 23) in Group D, the transportation, storage, and postal services sector (No. 24), the public services sector (No. 27), and the professional, scientific, and technical services sector (No. 28) in Group E. Although the direct energy consumption of sector No. 8 increased, its embodied energy consumption declined. The reason for this change remains a topic for future study. In contrast, the embodied energy consumption of sectors 13, 17, 19 increased largely from 1987 to 2007; however, their direct energy consumption showed a decline. A decrease in direct energy consumption with an increase of embodied energy consumption can be attributed to the ongoing industrial transition and updates in these sectors.

Another important indicator to analyze is the ratio of direct and indirect energy consumption by sectors, or the proportion of direct and indirect energy in embodied energy. We found that in almost all cases, the indirect energy consumption in a production process was higher than direct energy. The average ratio of direct energy consumption to total energy consumption for the 30 sectors under study was 33% in 1987 but 22% in 2007(see Table S1 and S2 in the appendix). The change of this ratio further illustrates the transition of Beijing's economy from a production-based and manufacturing dominated system to a consumption-based and service-dominated system. In other words, urban economic activities moved along the production chain and closer to the end-users. China emphasized the development of heavy industry from 1949 until the late 1970s. In response to the state industrialization strategy, Beijing built the first side-blown converter for the Shougang Iron and Steel Plant for steel production in 1958 and launched the Sinopec Beijing Yanshan Group in 1970. However, more recently the industrial structure has been undergoing a transformation from traditional heavy industries to modern high technology and high-value-added industries, a change which has been actively promoted by Beijing's municipal government. This transformation of the urban economy undoubtedly has had a great influence on the level of each sector's direct and indirect energy consumption and the distribution of energy consumption among sectors.

From the perspective of energy efficiency as indicated by energy intensity or energy consumption per unit of GDP, traditional heavy industries in Group C, such as nonmetallic production and metal production still remain as the most energy-intensive sectors in terms of direct energy intensity, although the energy efficiency of these sector has largely improved. For instance, the direct energy required for unit output of metal smelting and pressing has substantially decreased from 1.15E-03 tce/yuan in 1987 to 4.62E-04 tce/yuan tce in 2007. Despite of the decline, the energy demand per unit of GDP in petroleum processing and coking increased greatly, which can be ultimately attributed to the increasingly stringent environmental regulation (e.g., extra energy is needed for auxiliary equipment associated with pollutant treatment). Another interesting finding is that the service sector has become more energy efficient than it was 20 years ago. In general, there was not much change in the proportion of direct and indirect energy consumption of most of the energy-intensive sectors. This is because most of these sectors consumed energy in a direct form while the amount of indirect energy consumed during production was typically low. In contrast, there are great differences between direct and embodied energy intensity in some other sectors. For example, the construction sectors and professional, scientific, and technical services sectors, all of which have a relatively low direct energy intensity but required much higher indirect energy consumption.

### Transmission mechanism among economic sectors

**Figure 5:**
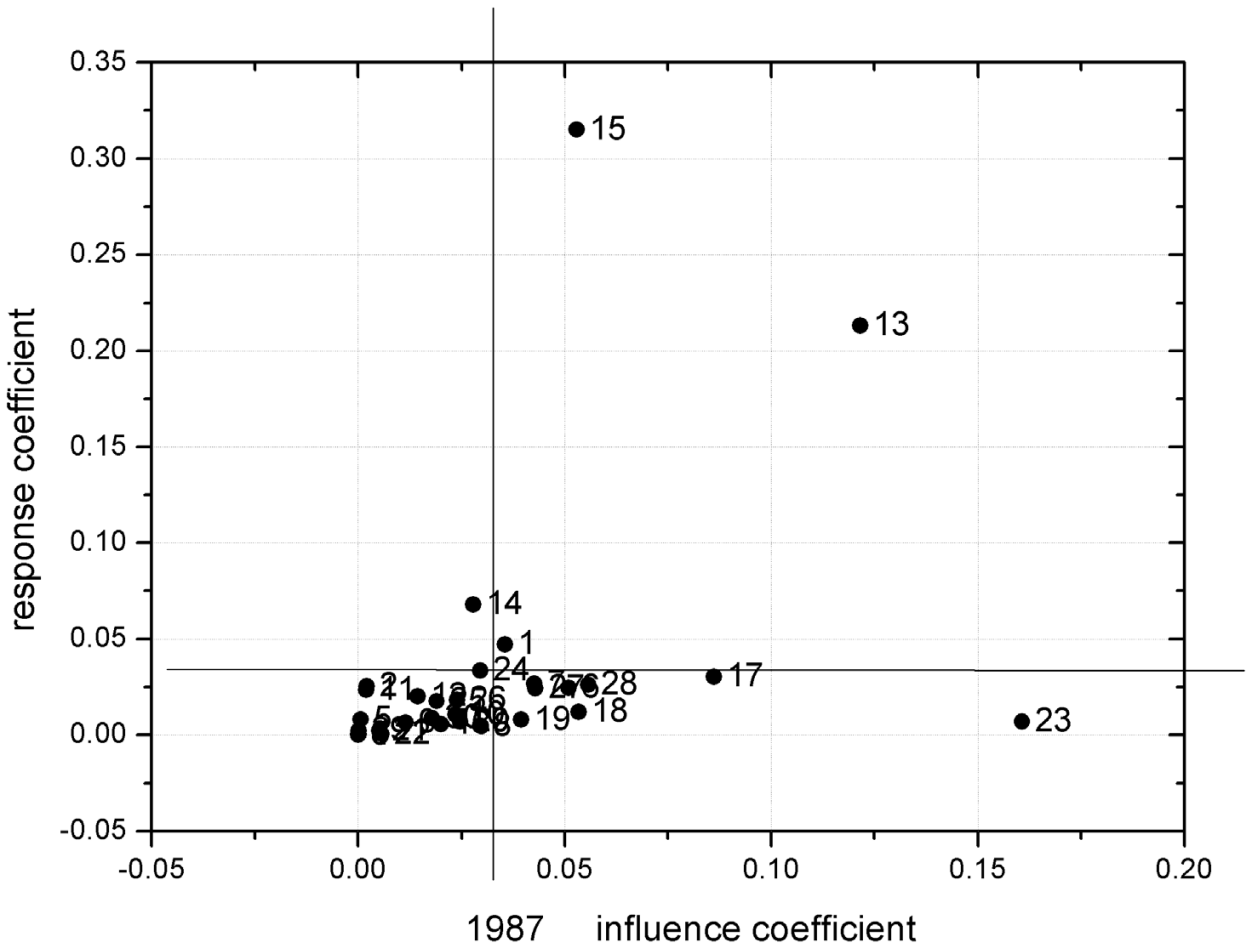
Planar graph of RC and IC of the 30 sectors in 1987 (see Table 2 for sector code definitions).

**Figure 6:**
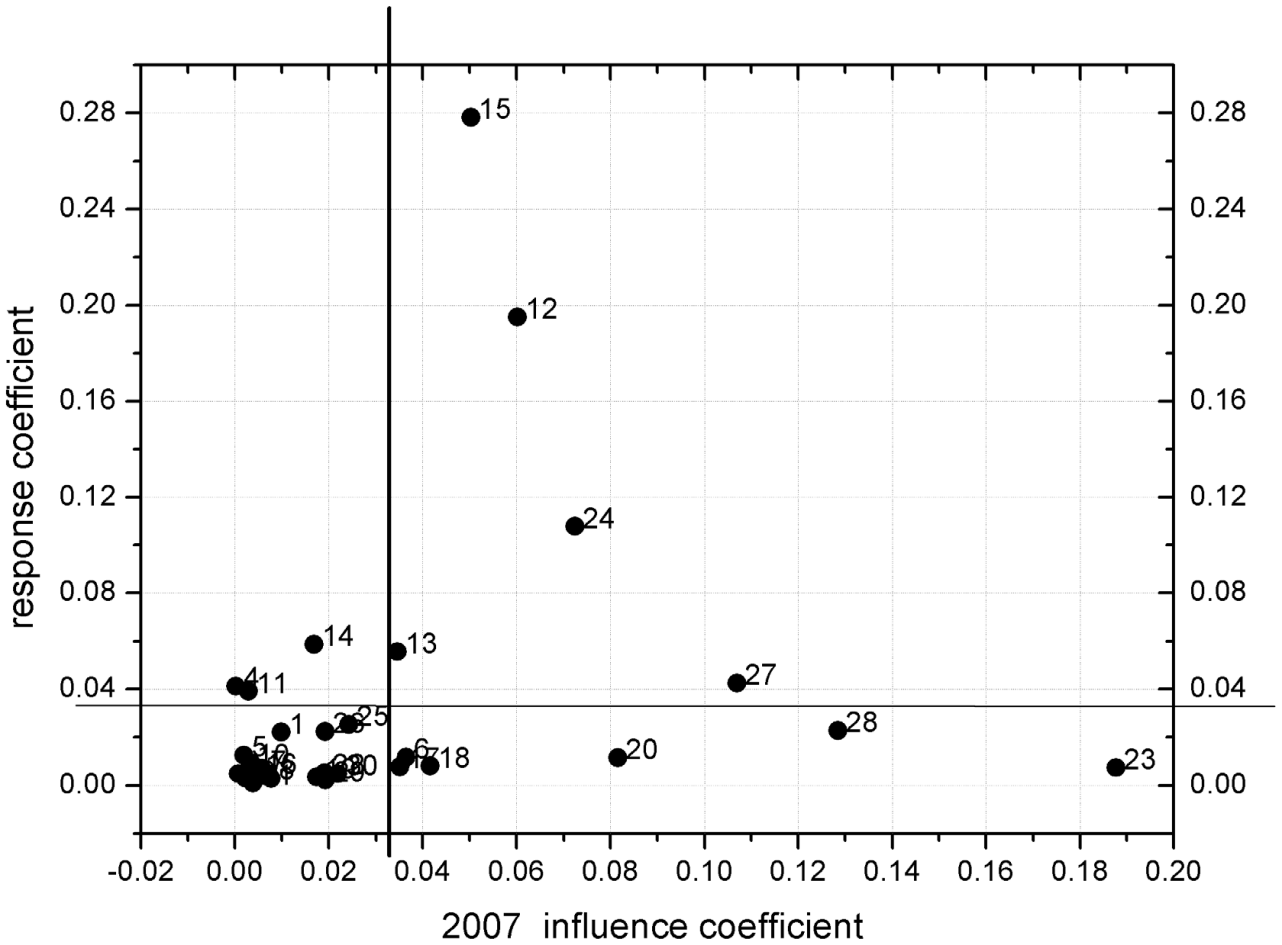
Planar graph of RC and IC of the 30 sectors in 2007 (see Table 2 for sector code definitions).

The IC and RC for different economic sectors in 1987 and 2007 were calculated and plotted in Figure 5 and Figure 6 in the form of a planar graph. The average values of the two indicators in 1987 and 2007 were 0.033 and 0.034, respectively. These average values were then used to define the starting point of the coordinate systems shown in Figure 5 and Figure 6. Sectors located in quadrant I are characterized by higher values of IC and RC, which implies that an increase in their final energy demand would have a great effect on other sectors' direct energy consumption. Conversely, these sectors themselves would also have been greatly affected by energy demand increases in other sectors. In other words, these sectors demonstrated a powerful ability to diffuse and to receive impacts. Because these sectors have both backward and forward linkages, they are usually regarded as key sectors in the energy system. Sectors located in quadrant II are characterized by higher RC and lower IC, which implies that their energy consumption is influenced by the demand of other sectors and thus have a strong forward linkage effect. In general, these sectors are usually in an upstream position in the production chain. They are responsible for providing embodied energy for the downstream sectors. Sectors located in quadrant III have lower-than-average RC and IC values, indicating that their energy consumption is less affected by other sectors. Sectors located in quadrant IV have relatively higher IC values and lower than average RC values. A change in their demand would therefore have a great effect on other sectors' energy consumption. These sectors are usually located in the downstream segment of the production chain and have backward linkages with other sectors.

As shown in Figure 5, in 1987 the three most important sectors were metal smelting and pressing (No. 15), chemicals (No. 13), and construction (No. 23). Sectors 15 and 13 in Group C had a much higher RC than other sectors, which means these two sectors had a much stronger forward linkage with other sectors, while sector 23 in Group D had a much higher IC, indicating that it was a key sector in backward linkages with other sectors in the energy system. Figure 6 shows that great changes have occurred in the interrelationships among sectors over the last two decades, a period corresponding to Beijing's rapid urbanization. In 2007, the sectors located in quadrant I with the closest correlations with other sectors were associated with not only sectors belonging to Group C, but also sectors in Group E. Not surprisingly, metal smelting and pressing sector (No. 15) and petroleum processing and coking sectors (No. 12) had a relatively higher RC and therefore a stronger forward linkage with other sectors, while sectors construction (No. 23), public service (No. 27) and professional, scientific, and technical services (No. 28) had a much higher IC, indicating a stronger driving force for energy consumption of other sectors. In addition, the transportation, storage, and postal services sector (No. 24) has strong backward and forward linkages with its upstream and downstream sectors. Nevertheless, sector 15 remains the sector with the largest forward linkages, while sector 23 has the largest backward linkages with another sector in both 1987 and 2007. It can be easily concluded that energy saving strategies should be taken from a supply chain perspective, not ender user perspective. Sectors like construction should also be considered as “energy intensive”, although their consumption is concentrated in the supply chain activity rather than their direct energy consumption [11].

Given this, transmission mechanism such as forward and backward linkages among sectors, are especially important for the development of energy policy. Specifically, the upstream and downstream sectoral collaboration along the whole supply chain is essential to establish policy mix for effective and efficient urban energy management. For those traditional heavy industries with high RC (for instance, sectors 15 and 12 in 2007), efforts should be focused on the improvement of efficiency through cleaner production, energy audits, technology updates, compulsory phase-out/shutdown of inefficient manufacturing facilities, capability-building programs on energy saving awareness, which would contribute to the improvement of embodied energy intensity of sectors in downstream; for those sectors with higher indirect energy consumptions, efforts should focus on addressing their supply chain energy consumption, such as greening their supply chain and controlling the irrational final demand.

### Concluding remarks

Over the last 20 years, China has undergone unprecedented rapid urbanization and profound structural changes in the urban economy. Recently the Chinese government has reemphasized urbanization as an important development strategy, and projects another 350 million more people to join the current Chinese urban population over the next decade. Such a large-scale urbanization will most likely spur energy demands for the construction of new buildings and infrastructure; additional residential energy uses will increase as well, as rural biomass is replaced with urban commercial energy services. To achieve the ambitious urbanization strategy, there is a need to adjust energy consumption behavior in order to meet the great challenges for energy demand and environmental protection. This would also have strong implications for the world energy market and climate change mitigation activities.

Beijing provides an example of the kind of change in energy consumption pattern associated with rapid urbanization. In this paper, we analyze the direct and indirect energy consumption for the whole city and by 30 economic sectors in Beijing during 1987–2007. Results show that total energy consumption has steadily increased despite a decline in overall energy intensity. At the sector level, we find that most of the sectors experienced a decrease in direct energy consumption, but an increase in indirect energy consumption, especially the construction, transportation and service sectors. Changes in the pattern of energy consumption have shown that Beijing has shifted from a production-based and manufacturing-dominated system to a consumption-based and service-dominated system.

Analysis in this paper demonstrates that the traditional way of analyzing urban energy consumption, which focuses solely on direct energy consumption, ignores the complex energy flows among sectors and is insufficient for making robust energy policies. Energy saving measures and efficiency improvement policies should not only consider those traditional heavy industries, but should also pay attention to other sectors along the supply chain. For instance, greater attention should be paid to the construction sectors and the professional, scientific, and technical services sectors, which demonstrated relatively low direct energy intensity, but demanded much higher indirect energy consumption. It is well known that China's urban economy, such as Beijing, has been driven primarily by infrastructure construction and capital investment. Shifting investment to areas such as education and technological innovation would be helpful not only for long-term economic growth but also for achieving energy security policy objectives.

In summary, an economy-wide system accounting analysis allows us to trace the direct, as well as indirect energy consumption along the supply chains of an economy. It is therefore a useful tool for systematic policy making. Our analysis highlights that it is embodied energy as opposed to direct energy that provides a holistic picture on urban economy's energy consumption. Correspondingly, energy saving and efficiency improvement policy should be based on such a system accounting approach, e.g., enhancing end treatment for those sectors with high RC, while addressing source control and supply chain management for those sectors with higher IC.

## Supporting Information

Table S1 **Direct sectoral energy consumption associated with the concerned 9 years (unit: Mtce).**
(DOCX)

Table S2 **Embodied energy consumption by sectors associated with the concerned 9 years (unit: Mtce).**
(DOCX)
