# Supplementary material for: Input-Output Modeling for Urban Energy Consumption in Beijing: Dynamics and Comparison
Source: PLoS One. 2014 Mar 3;9(3):e89850. doi: 10.1371/journal.pone.0089850 (PMC3940614; doi:10.1371/journal.pone.0089850)
Supplement: Table S1 — Direct sectoral energy consumption associated with the concerned 9 years (unit: Mtce). (DOCX) [file pone.0089850.s001.docx]

Table S1. Direct sectoral energy consumption associated with the concerned 9 years (unit: Mtce)

| Sector code | 1987 | 1990 | 1992 | 1995 | 1997 | 2000 | 2002 | 2005 | 2007 |
| --- | --- | --- | --- | --- | --- | --- | --- | --- | --- |
| 1 | 0.84 | 1.26 | 1.59 | 1.18 | 0.96 | 1.05 | 1.03 | 0.86 | 0.96 |
| 2 | 0.13 | 0.13 | 0.16 | 0.17 | 0.14 | 0.13 | 0.06 | 0.08 | 0.07 |
| 3 | 0.00 | 0.00 | 0.00 | 0.00 | 0.00 | 0.00 | 0.00 | 0.00 | 0.02 |
| 4 | 0.00 | 0.01 | 0.02 | 0.02 | 0.01 | 0.03 | 0.05 | 0.12 | 0.10 |
| 5 | 0.06 | 0.07 | 0.08 | 0.04 | 0.09 | 0.11 | 0.02 | 0.21 | 0.26 |
| 6 | 0.48 | 0.61 | 0.64 | 0.66 | 0.69 | 0.67 | 0.77 | 0.95 | 0.97 |
| 7 | 0.43 | 0.45 | 0.45 | 0.41 | 0.42 | 0.30 | 0.22 | 0.20 | 0.16 |
| 8 | 0.15 | 0.10 | 0.11 | 0.11 | 0.14 | 0.12 | 0.11 | 0.19 | 0.19 |
| 9 | 0.15 | 0.12 | 0.12 | 0.10 | 0.07 | 0.05 | 0.09 | 0.12 | 0.11 |
| 10 | 0.27 | 0.30 | 0.31 | 0.24 | 0.29 | 0.28 | 0.26 | 0.39 | 0.40 |
| 11 | 0.34 | 0.42 | 0.50 | 0.68 | 0.86 | 1.22 | 2.43 | 2.60 | 2.50 |
| 12 | 0.45 | 0.63 | 3.87 | 4.01 | 4.16 | 4.45 | 6.28 | 6.64 | 6.93 |
| 13 | 4.72 | 4.94 | 2.03 | 5.44 | 5.47 | 2.91 | 1.89 | 2.55 | 2.62 |
| 14 | 1.75 | 1.60 | 1.68 | 1.58 | 1.87 | 2.39 | 2.22 | 2.99 | 3.12 |
| 15 | 4.21 | 4.58 | 5.53 | 7.27 | 9.01 | 9.17 | 8.69 | 6.68 | 6.84 |
| 16 | 0.17 | 0.20 | 0.21 | 0.13 | 0.13 | 0.17 | 0.18 | 0.24 | 0.25 |
| 17 | 0.80 | 0.76 | 0.64 | 0.48 | 0.47 | 0.50 | 0.37 | 0.54 | 0.66 |
| 18 | 0.31 | 0.32 | 0.35 | 0.52 | 0.48 | 0.38 | 0.39 | 0.64 | 0.68 |
| 19 | 0.17 | 0.16 | 0.16 | 0.14 | 0.13 | 0.14 | 0.16 | 0.14 | 0.16 |
| 20 | 0.13 | 0.13 | 0.14 | 0.17 | 0.15 | 0.19 | 0.28 | 0.58 | 0.61 |
| 21 | 0.05 | 0.05 | 0.04 | 0.05 | 0.04 | 0.05 | 0.04 | 0.05 | 0.06 |
| 22 | 0.01 | 0.01 | 0.01 | 0.36 | 0.32 | 0.03 | 0.07 | 0.15 | 0.13 |
| 23 | 0.28 | 0.37 | 0.40 | 0.35 | 0.34 | 0.68 | 0.89 | 1.03 | 1.09 |
| 24 | 0.80 | 1.38 | 1.66 | 1.22 | 1.81 | 3.13 | 3.99 | 5.63 | 8.41 |
| 25 | 0.41 | 0.97 | 1.39 | 0.86 | 0.70 | 0.86 | 0.69 | 1.58 | 2.03 |
| 26 | 0.71 | 0.52 | 0.72 | 0.40 | 0.59 | 0.57 | 1.34 | 2.00 | 2.50 |
| 27 | 0.90 | 0.90 | 0.67 | 0.86 | 1.52 | 2.34 | 3.54 | 5.63 | 6.17 |
| 28 | 0.93 | 1.10 | 1.07 | 1.88 | 1.74 | 2.18 | 2.29 | 3.25 | 3.57 |
| 29 | 0.05 | 0.14 | 0.12 | 0.37 | 0.41 | 0.23 | 0.30 | 0.28 | 0.34 |
| 30 | 0.34 | 0.56 | 0.44 | 0.62 | 0.59 | 0.76 | 0.54 | 0.82 | 0.88 |
