# Supplementary material for: Input-Output Modeling for Urban Energy Consumption in Beijing: Dynamics and Comparison
Source: PLoS One. 2014 Mar 3;9(3):e89850. doi: 10.1371/journal.pone.0089850 (PMC3940614; doi:10.1371/journal.pone.0089850)
Supplement: Table S2 — Embodied energy consumption by sectors associated with the concerned 9 years (unit: Mtce). (DOCX) [file pone.0089850.s002.docx]

Table S2. Embodied energy consumption by sectors associated with the concerned 9 years (unit: Mtce)

| Sector code | 1987 | 1990 | 1992 | 1995 | 1997 | 2000 | 2002 | 2005 | 2007 |
| --- | --- | --- | --- | --- | --- | --- | --- | --- | --- |
| 1 | 1.50 | 2.43 | 3.08 | 3.10 | 2.67 | 2.72 | 3.29 | 2.50 | 2.81 |
| 2 | 0.22 | 0.29 | 0.27 | 0.24 | 0.25 | 0.26 | 0.50 | 0.87 | 1.09 |
| 3 | 0.00 | 0.00 | 0.00 | 0.00 | 0.00 | 0.00 | 0.00 | 0.00 | 0.15 |
| 4 | 0.02 | 0.08 | 0.17 | 0.08 | 0.07 | 0.08 | 0.10 | 0.24 | 0.16 |
| 5 | 0.19 | 0.19 | 0.48 | 0.11 | 0.21 | 0.35 | 0.29 | 0.48 | 0.59 |
| 6 | 1.88 | 3.03 | 3.31 | 3.72 | 4.22 | 3.20 | 3.48 | 3.72 | 5.19 |
| 7 | 1.78 | 2.77 | 1.89 | 1.57 | 1.46 | 0.96 | 0.94 | 0.77 | 0.61 |
| 8 | 1.15 | 1.65 | 2.05 | 1.34 | 1.19 | 0.94 | 0.68 | 0.90 | 0.79 |
| 9 | 0.55 | 0.67 | 0.59 | 0.57 | 0.68 | 0.70 | 0.59 | 0.49 | 0.88 |
| 10 | 1.80 | 2.55 | 2.22 | 1.35 | 1.19 | 1.26 | 1.46 | 1.53 | 2.09 |
| 11 | 0.63 | 0.97 | 1.61 | 1.30 | 1.37 | 1.97 | 3.15 | 6.34 | 9.53 |
| 12 | 0.96 | 1.37 | 5.04 | 5.81 | 4.91 | 6.60 | 9.89 | 11.00 | 10.89 |
| 13 | 8.02 | 9.51 | 6.35 | 11.60 | 12.48 | 7.84 | 8.60 | 7.02 | 10.30 |
| 14 | 2.87 | 3.21 | 3.31 | 3.15 | 3.82 | 4.28 | 4.71 | 5.13 | 7.17 |
| 15 | 6.41 | 8.14 | 10.01 | 11.96 | 14.74 | 12.92 | 14.60 | 11.18 | 16.50 |
| 16 | 1.90 | 2.61 | 3.10 | 1.83 | 2.51 | 2.76 | 3.39 | 2.11 | 4.73 |
| 17 | 3.98 | 5.14 | 3.80 | 3.51 | 3.91 | 3.35 | 5.95 | 4.33 | 10.17 |
| 18 | 2.25 | 3.26 | 3.62 | 5.16 | 3.87 | 3.13 | 5.39 | 7.27 | 9.90 |
| 19 | 1.58 | 2.00 | 1.42 | 1.10 | 1.59 | 1.66 | 2.65 | 1.77 | 3.84 |
| 20 | 1.04 | 1.47 | 2.07 | 3.04 | 4.74 | 8.97 | 10.98 | 11.22 | 18.71 |
| 21 | 0.21 | 0.29 | 0.25 | 0.46 | 0.39 | 0.43 | 0.93 | 0.91 | 1.64 |
| 22 | 0.32 | 0.58 | 1.10 | 0.45 | 1.11 | 0.47 | 0.26 | 0.85 | 1.12 |
| 23 | 6.25 | 7.26 | 6.15 | 9.39 | 13.72 | 20.63 | 26.43 | 24.08 | 31.99 |
| 24 | 1.76 | 3.09 | 2.69 | 6.04 | 6.27 | 8.29 | 8.66 | 12.45 | 23.11 |
| 25 | 1.01 | 1.74 | 6.50 | 1.57 | 2.20 | 3.22 | 2.45 | 5.92 | 7.28 |
| 26 | 1.02 | 0.63 | 1.67 | 0.82 | 1.31 | 1.44 | 3.18 | 4.52 | 5.87 |
| 27 | 2.19 | 2.77 | 3.56 | 3.74 | 2.15 | 11.16 | 13.70 | 24.38 | 29.58 |
| 28 | 2.77 | 3.32 | 5.27 | 7.78 | 6.18 | 9.22 | 12.63 | 17.17 | 30.83 |
| 29 | 0.11 | 0.32 | 1.57 | 7.14 | 6.12 | 3.52 | 4.51 | 2.31 | 5.07 |
| 30 | 0.69 | 1.25 | 1.70 | 2.16 | 2.13 | 2.61 | 2.41 | 3.12 | 4.31 |
